# Metabolic engineering for high yielding L(-)-carnitine production in *Escherichia coli*

**DOI:** 10.1186/1475-2859-12-56

**Published:** 2013-05-29

**Authors:** Paula Arense, Vicente Bernal, Daniël Charlier, José Luis Iborra, Maria Remedios Foulquié-Moreno, Manuel Cánovas

**Affiliations:** 1 Department of Biochemistry and Molecular Biology B and Immunology, Faculty of Chemistry, University of Murcia, Campus de Espinardo. Regional Campus of International Excellence “Campus Mare Nostrum”, P.O. Box 4021, Murcia E-30100, Spain; 2 Research group of Microbiology, Vrije Universiteit Brussel, Pleinlaan 2, B-1050, Brussels, Belgium; 3 Laboratory of Molecular Cell Biology, KU Leuven. VIB (Flanders Institute of Biotechnology), Kasteelpark Arenberg, 31; bus 2438, Heverlee 3001, Belgium; 4 Current address: Grupo de Bioenergía. Dirección de Tecnología, Centro de Tecnología de REPSOL, Carretera A-5, Km 18, Móstoles-Madrid, 28935, Spain

**Keywords:** Biotransformation, Carnitine, Strain engineering, Artificial promoter, Knock-in, Knock-out, Crotonobetaine

## Abstract

**Background:**

L(-)-carnitine production has been widely studied because of its beneficial properties on various diseases and dysfunctions. Enterobacteria possess a specific biotransformation pathway which can be used for the enantioselective production of L(-)-carnitine. Although bioprocesses catalyzed by enzymes or whole cells can overcome the lack of enantioselectivity of chemical methods, current processes for L(−)-carnitine production still have severe disadvantages, such as the low yields, side reactions and the need of high catalyst concentrations and anaerobic conditions for proper expression of the biotransformation pathway. Additionally, genetically engineered strains so far constructed for L(-)-carnitine production are based on plasmids and, therefore, suffer from segregational unstability.

**Results:**

In this work, a stable, high yielding strain for L(-)-carnitine production from low cost substrates was constructed. A metabolic engineering strategy was implemented in a multiple mutant for use in both growing and resting cells systems. The effect of mutations on gene expression and metabolism was analyzed to characterize the productivity constraints of the wild type and the overproducer strains. Precise deletion of genes which encode proteins of central and carnitine metabolisms were performed. Specifically, flux through the TCA cycle was increased by deletion of *aceK* (which encodes a bifunctional kinase/phosphatase which inhibits isocitrate dehydrogenase activity) and the synthesis of the by-product γ-butyrobetaine was prevented by deletion of *caiA* (which encodes a crotonobetainyl-CoA reductase). Both mutations led to improve the L(-)-carnitine production by 20 and 42%, respectively. Moreover, the highly regulated promoter of the *cai* operon was substituted by a constitutive artificial promoter increasing the biotransformation rate, even under aerobic conditions. Resting cells of the BW Δ*aceK* Δ*caiA* p37*cai* strain produced 59.6 mmol l^-1^ · h^-1^ of L(−)-carnitine, doubling the productivity of the wild type strain. In addition, almost total conversion was attained in less than two hours without concomitant production of the side product γ–butyrobetaine.

**Conclusions:**

L(-)-carnitine production has been enhanced by strain engineering. Metabolic engineering strategies herein implemented allowed obtaining a robust and high yielding *E. coli* strain. The new overproducer strain attained almost complete conversion of crotonobetaine into L(-)-carnitine with growing and resting cells, and even under aerobic conditions, overcoming the main environmental restriction to carnitine metabolism expression. So far, this is the best performing L(-)-carnitine production *E. coli* strain described.

## Background

Worldwide, the demand of L(-)-carnitine [R(−)-3-hydroxy-4-trimethylaminobutyrate] is increasing due to its multiple applications as pharmaceutical and nutraceutical product, hence the need of developing more efficient production methods. Chemical synthesis yields a racemic mixture of D,L-carnitine, which cannot be administered to patients [1-4]. The natural enantioselectivity of microbial and enzymatic biotransformations offers an advantage over classical chemical synthesis. Several biological processes have been developed for the production of L(-)-carnitine from non-chiral precursors [5-12] especially using strains belonging to the genera *Escherichia* and *Proteus.* At the industrial level*,* Lonza belongs a proprietary strain of a non-disclosed genus branching between *Agrobacterium* and *Rhizobium* and close to *Rhizobium meliloti*[13]*.*

**Figure 1:**
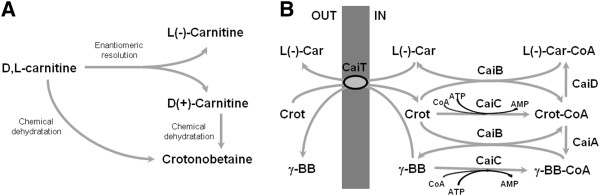
**Synthesis of L(-)-carnitine.** (**A**) Chemical synthesis of L(-)-carnitine and crotonobetaine. (**B**) Metabolism of trimethylammonium compounds in *E. coli*. Biotransformation of crotonobetaine into L(-)-carnitine. Abbreviations: L(-)-Car: L(-)-carnitine; Crot: crotonobetaine; γ-BB: γ-butyrobetaine; CaiT: L(-)-carnitine/crotonobetaine/γ-butyrobetaine protein transporter; CaiB: crotonobetainyl-CoA:L(−)-carnitine CoA-tranferase; CaiC: L(-)-carnitine, crotonobetaine or γ-butyrobetaine CoA-ligase; CaiD: crotonobetainyl-CoA hydratase. Adapted from [6].

Crotonobetaine (dehydrated D,L-carnitine) and D(+)-carnitine are by-products from the chemical L(-)-carnitine production process (Figure 1A), which can be transformed into L(-)-carnitine. This enantioselective biotransformation has the potential to enhance the overall economic and environmental viability of the chemical synthesis process. In this respect, the L(-)-carnitine metabolism in *E. coli* has been widely studied and characterized [5,6,14-16] because of its role in anaerobic respiration, and stress survival, especially in osmoprotection [17-19]. *E. coli* is able to transform crotonobetaine into L(-)-carnitine through a series of sequential steps. Substrates and products are transported by a specific membrane antiporter (CaiT) [20]. All biochemical steps occur at the level of coenzyme A thioesters: activation of betaines involves an ATP-dependent CoA-ligase (CaiC), and a crotonobetainyl-CoA:carnitine CoA-transferase (CaiB) which inexpensively exchanges the CoA moiety between betaines [16,21-23]. The enantioselective hydration is catalyzed by a crotonobetainyl-CoA hydratase (CaiD) [16,22]. As a side reaction, crotonobetaine can be reduced to γ-butyrobetaine by means of a crotonobetainyl-CoA reductase (CaiA) [16,24], a respiration process which is inhibited by electron acceptors such as oxygen or fumarate (Figure 1B). All these activities are encoded by two divergent operons: *caiTABCDE*, which encodes the carnitine biotransformation enzymes [25], and *fixABCX*, which encodes putative flavoproteins involved in anaerobic carnitine respiration [26,27]. Both are expressed from a common intergenic promoter-operator region, which is tightly regulated by cAMP-CRP, FNR, and the specific transcriptional activator CaiF [28]. Expression of *caiF* is activated by cAMP-CRP and FNR, which regulates the expression of hundreds of genes under anaerobic conditions [29-31]. So far, carnitine production by *E. coli* is carried out in anaerobic conditions to induce the expression of the *cai* operon, as described in the current model of regulation [5,6,15].

With the aim of developing a combined and sustainable chemical-biotechnological process for industrial production of L(-)-carnitine, we have previously reported several strategies to enhance the biotransformation of inexpensive substrates such as crotonobetaine or D(+)-carnitine. Previous approaches focused on the use of high cell density cultures, immobilized or resting cells [11,14,32]. Up to 40-60% crotonobetaine conversion was obtained with the wild type, non pathogenic, *E. coli* O44K74 strain [32,33], and 60-70% using recombinant *E. coli* strains overexpressing either the carnitine-CoA ligase or the crotonobetainyl-CoA hydratase genes (encoded by *caiC* and *caiD*, respectively) [34,35].

The major drawbacks of previous processes are the low conversion yields and the production of the side-product γ-butyrobetaine. Despite high volumetric productivities, the conversion yield could be improved, since the presence of excess (non-transformed) substrates and by-products in the biotransformation medium seriously hinders downstream processing. Moreover, the *cai/fix* operons are only expressed under anaerobic conditions, with a concomitantly decreased energetic efficiency, and the need to supplement the medium with fumarate to inhibit the carnitine respiration pathway. In addition, using plasmid-transformed strains in large scale cultivation presents several drawbacks such as the dependence on expensive inducers and antibiotics. Moreover, plasmids can be lost as a result of inefficient segregation between daughter cells and the high metabolic burden imposed by the maintenance of this extra genetic material [14,36]. Overall, all these constraints further restrict the economics of the bioprocess, preventing its implementation in an industrial scale.

This work aims at improving L(-)-carnitine production in *E. coli* by strain engineering techniques, overcoming the major drawbacks previously exposed. All modifications were performed at the chromosomal level in order to obtain genetically stable, marker-free, high-yielding strains.

## Results

### Strain engineering for L(-)-carnitine production

**Figure 2:**
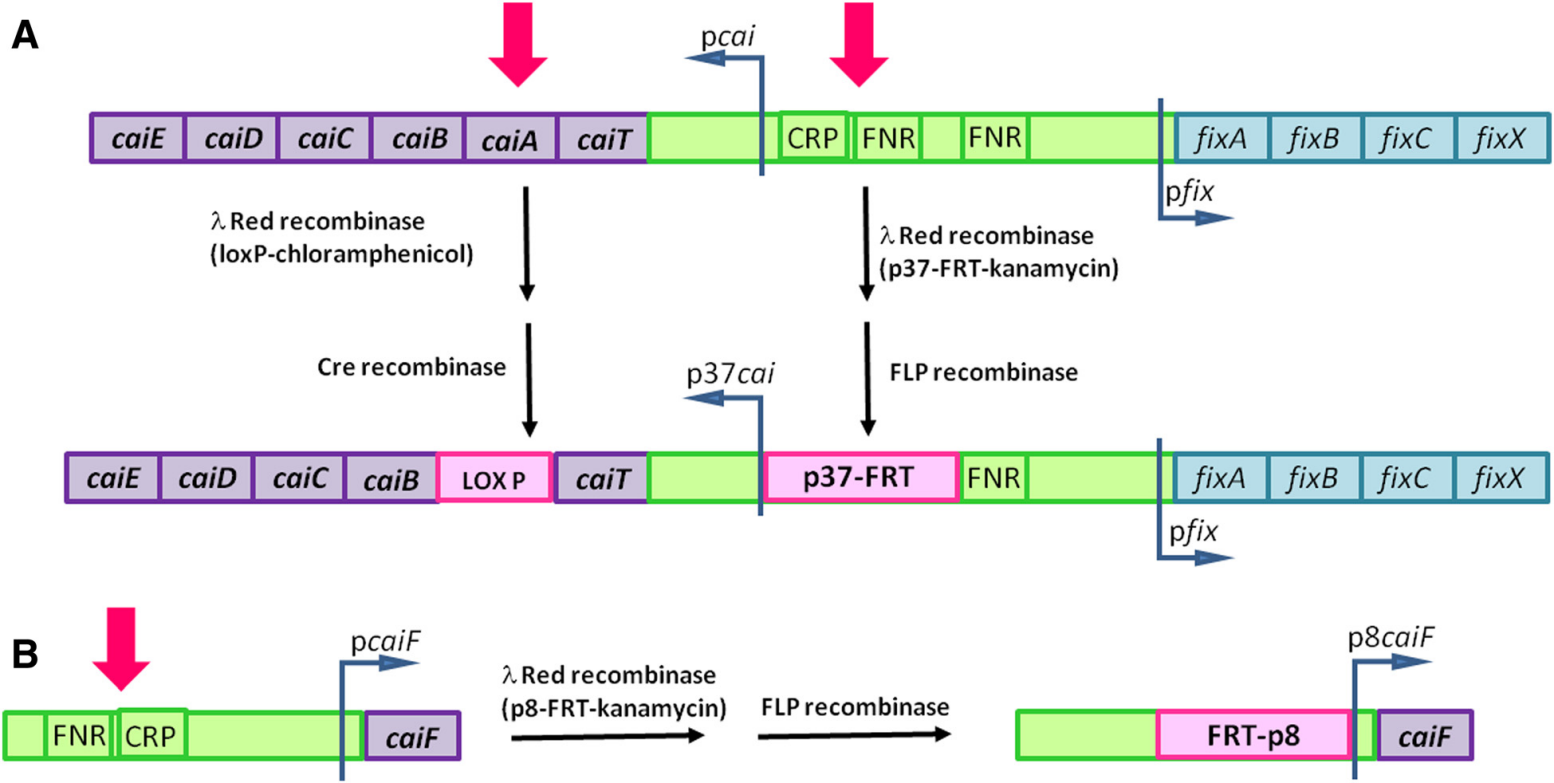
**Strain engineering strategy followed to improve L(−)-carnitine production in *E. coli *BW25113.** (**A**) Deletion of the *caiA* gene and replacement of the endogenous *cai* operon promoter and regulatory sequences (FNR and CRP binding sites) by the p37 artificial promoter. (**B**) Replacement of the endogenous *caiF* promoter and a close regulatory region (FNR and CRP binding sites) by the p8 artificial promoter.

On the basis of previous knowledge, three strategies were designed to enhance carnitine production, dealing with either central or secondary metabolism: (i) altering the glyoxylate shunt/TCA cycle flux ratio at the isocitrate node, (ii) avoiding the reduction of crotonobetaine to γ-butyrobetaine (carnitine respiration pathway), and (iii) enhancing the expression of the L(-)-carnitine operon structural (*caiTABCDE*) or regulatory genes (*caiF*) to relieve repression by aerobic conditions (Figure 2). All modifications were performed in *E. coli* BW25113, in which L(-)-carnitine productivity is in the same order of magnitude as in the well characterized *E. coli* O44K74 strain. To determine the effect on L(-)-carnitine production, the strains were cultured anaerobically in LB-CB medium.

**Figure 3:**
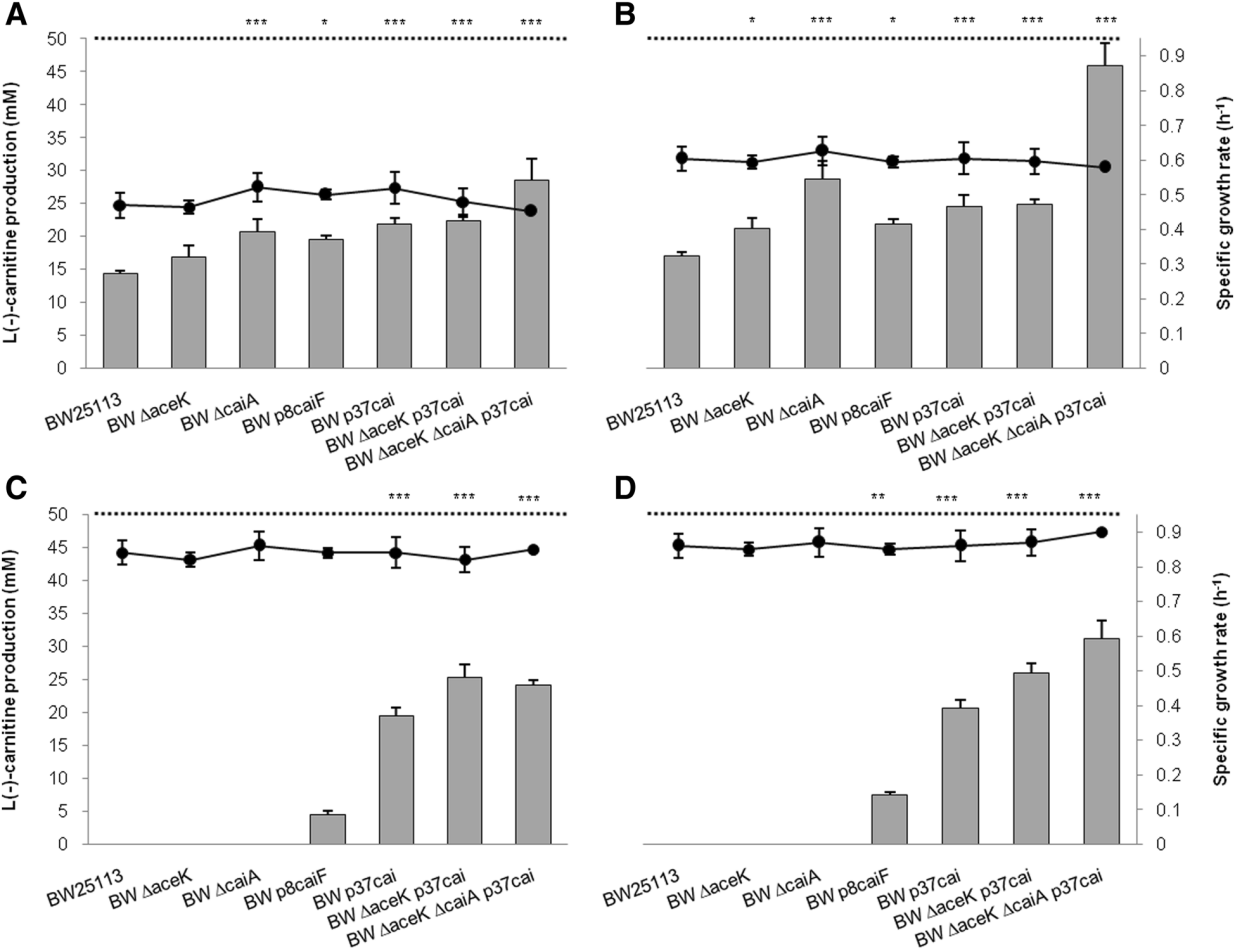
**Effect of the mutations of central and secondary metabolism on L(-)-carnitine production.** Experiments were performed in LB medium supplemented with 50 mM crotonobetaine (LB-CB) in the absence and presence of fumarate and under aerobic and anaerobic conditions. Anaerobic cultures: (**A**) LB-CB medium and (**B**) LB-CB medium supplemented with 12.5 mM fumarate. Aerobic cultures: (**C**) LB-CB medium and (**D**) LB-CB medium supplemented with 12.5 mM fumarate. Bars represent the L(-)-carnitine yield after 24 h and dots represent the specific growth rate. Discontinuous line indicates the maximum production (corresponding to 50 mM L(−)-carnitine). Adjusted p-values for ANOVA (p < 0.001) and Tukey test of L(-)-carnitine production, *p < 0.05, **p < 0.01, ***p < 0.001.

First, as regards the modification of central metabolism, the genes encoding isocitrate lyase (*aceA*) and isocitrate dehydrogenase phosphatase/kinase (*aceK*) were deleted. The *aceK* knockout strain (devoid of post-translational control of isocitrate dehydrogenase) showed a modest improvement in the production of L(-)-carnitine (20%), while deletion of *aceA* (encoding the first enzyme of the glyoxylate shunt) only had a slight effect (6%) (Figure 3). No further improvement was observed in the *aceAK* double mutant.

Second, to avoid the reduction of crotonobetainyl-CoA into γ-butyrobetainyl-CoA, the *caiA* gene (encoding the crotonobetainyl-CoA reductase) was deleted (Figure 1), leading to an improvement in L(-)-carnitine production of 42% (Figure 3). The side-reaction was effectively suppressed since no γ-butyrobetaine was detected in the supernatant of any of the Δ*caiA* strains assayed (results not shown).

Furthemore, the expression of the carnitine metabolism structural (*cai* operon) and regulatory (*caiF*) genes was tuned using artificial promoters (Figure 2), eliminating the repression by aerobic conditions. When the endogenous promoters were replaced by constitutive artificial promoters, L(-)-carnitine production increased 51% and 20% in the BW p37*cai* and BW p8*caiF* strains, respectively (Figure 3).

Altogether, statistically significant differences in the specific L(-)-carnitine production rates were observed between the assayed strains. The highest L(-)-carnitine titers were achieved by the BW p37*cai* and BW ∆*caiA* strains (21.7 and 20.7 mM respectively) (Figure 3A).

To further study these strains with single modifications, growth and carnitine production rates were also determined in anaerobic cultures in the presence of fumarate (Figure 3B) (used as an alternative electron acceptor [24,33]). The BW ∆*caiA* strain reached the highest production, 28.7 mM (p < 0.001) (Figure 3B) and a similar 20-30% increase in the specific growth rate was observed for all the strains with single modifications.

To further enhance productivity, all modifications that positively affected L(−)-carnitine production, namely, deletion of *aceK* and *caiA* and replacement of the *cai* promoter, were implemented in the same strain. Given its close proximity to the *cai* operon promoter, the *caiA* gene was deleted combining FRT and loxP sites in order to avoid the deletion of the contiguous *caiT* gene (Figure 2). As expected, the mutations did not affect growth significantly. Nevertheless, the specific L(-)-carnitine production rate was higher than in the single mutants. In order to check the effect of the mutations in the BW Δ*aceK* Δ*caiA* p37*cai* strain compared to the wild type, expression of genes belonging to the *cai/fix* operons was analyzed by qRT-PCR. Upstream (*caiT*) and downstream (*caiB* and *caiC*) genes of the deleted *caiA* were analyzed in order to assess whether a polar effect appeared due to this deletion. As expected, the constitutive promoter increased the expression of the carnitine metabolism genes. However, *caiB* and *caiC* exhibited a lower level of expression in the ∆*caiA* strains (Table 1), indicating that *caiA* deletion exerts a polar effect. In addition, the p37 promoter also enhanced expression of the *fix* operon (Table 1). This finding is not surprising, since the *cai* and *fix* operons are expressed from a common intergenic control region [29].

**Table 1:** Relative gene expression in the engineered strains growing anaerobically on LB-CB medium supplemented with 12.5 mM fumarate

**Strains**	***caiT***	***caiB***	***caiC***	***fixA***
BW ∆*caiA*	1.443 ± 0.20	0.86 ± 0.21	0.81 ± 0.06	1.32 ± 0.19
BW ∆*aceK* p37*cai*	3.00 ± 0.16	3.08 ± 0.24	2.18 ± 0.20-	1.95 ± 0.17
BW ∆*aceK* ∆*caiA* p37*cai*	3.23 ± 0.17	1.76 ± 0.10	1.47 ± 0.12	1.84 ± 0.10

For each gene, the transcription level of that gene in the wild type strain was used as reference to normalize the data. Relative gene expression in the wild type strain is, therefore, taken as 1. The results are the averages of three independent measurements of each gene/condition in three independent experiments.

Furthermore, the single (BW Δ*aceK* and BW Δ*caiA*), double (BW Δ*aceK* p37*cai*), and triple (BW Δ*aceK* Δ*caiA* p37*cai*) mutants and the wild type strain were grown in the absence (Figure 3A) and presence of fumarate (Figure 3B) under anaerobic conditions in LB-CB medium (Figure 3A, B). The combination of all three mutations contributed to the highest increase of specific L(-)-carnitine production rate (which doubled both in absence and presence of fumarate) and yield (reaching 70% and 92% of conversion, respectively). The highest titer obtained was 46 mM L(-)-carnitine with the BW Δ*aceK* Δ*caiA* p37*cai* strain (Table 2, Figure 3B).

**Table 2:** **Metabolic performance of wild-type (BW25113) and engineered (BW Δ*aceK *Δ*caiA *p37 *cai *) *E. coli *strains during L(−)-carnitine production in anaerobic and aerobic conditions**

**Strain and conditions**	**q**_**L-car**_	**q**_**Suc**_	**-q**_**Fum**_	**q**_**Acet**_	**q**_**EtOH**_	**q**_**Form**_
**Anaerobic cultures**						
LB-CB						
BW25113	9.30 ± 0.12	8.43 ± 0.22	---	15.71 ± 0.28	7.23 ± 0.20	4.21 ± 0.14
BW ∆*aceK* ∆*caiA* p37*cai*	18.87 ± 0.23***	7.20 ± 0.17***	---	17.44 ± 0.16***	2.49 ± 0.12***	12.96 ± 0.42***
LB-CB + fumarate (12.5 mM)						
BW25113	14.23 ± 0.15	13.89 ± 0.54	17.74 ± 0.26	13.15 ± 0.36	4.8 ± 0.32	N.D.
BW ∆*aceK* ∆*caiA* p37*cai*	27.75 ± 0.17***	10.31 ± 0.32***	19.51 ± 0.41***	16.44 ± 0.21***	3.95 ± 0.27***	N.D.
**Aerobic cultures**						
LB-CB						
BW25113	---	N.D.	---	9.49 ± 0.23	0.43 ± 0.05	---
BW ∆*aceK* ∆*caiA* p37*cai*	6.15 ± 0.10***	N.D.	---	7.79 ± 0.12***	1.17 ± 0.08*	---
LB-CB + fumarate (12.5 mM)						
BW25113	---	3.32 ± 0.11	9.42 ± 0.24	5.74 ± 0.10	0.15 ± 0.02	---
BW ∆*aceK* ∆*caiA* p37*cai*	7.02 ± 0.07***	2.95 ± 0.08	6.82 ± 0.17***	8.31 ± 0.14***	1.26 ± 0.06***	---

Specific production/consumption rates of L(−)-carnitine and the main extracellular metabolites were calculated during the early exponential phase of cultures. All rates are expressed in mmol g^-1^ h^-1^. Pairwise statistical comparison of the parameters assessed for both strains was perfomed with ANOVA and Tukey tests. Adjusted p-values are indicated as follows: *p < 0.05, **p < 0.01, ***p < 0.001.

### Biotransformation under aerobic conditions

As described before, it might be desirable to produce L(-)-carnitine under aerobic conditions with engineered strains that constitutively overexpressed the *caiF* gene and the *cai* operon. Tuning gene expression with artificial oxygen-independent promoters should allow reaching this goal and overcoming the limitations exhibited by the wild type strain [37,38].

Indeed, the engineered strains produced L(-)-carnitine under aerobic conditions when either the endogenous promoters p*caiF* or p*cai* were replaced by the constitutive promoters p8 or p37, respectively. As expected, the wild type, BW ∆*aceK,* and BW ∆*caiA* strains were not able to produce L(-)-carnitine (Figure 3C, D). The presence of fumarate did not affect the maximum specific growth rate, being 0.84 h^-1^ for cultures without fumarate and 0.86 h^-1^ for cultures supplemented with fumarate, although enhanced L(−)-carnitine production as in anaerobic cultures (Figure 3C and D). The L(−)-carnitine yield was higher in the strains that harbored the p37-promoter upstream the *cai* operon. Nevertheless, the activity of the promoter p8 was sufficient to activate the expression of carnitine metabolism and to produce L(-)-carnitine aerobically in the BW p8*caiF* strain. For p37-mutants, the biotransformation yield ranged between 40-60%. In spite of the fact that the strain with the highest specific carnitine production rate was BW ∆*aceK* p37*cai*, the strain BW ∆*aceK* ∆*caiA* p37*cai* showed the highest L(-)-carnitine yield when reached the stationary phase in the presence of fumarate (p < 0.001), obtaining 31.2 mM L(-)-carnitine. Altogether, the modifications performed allowed aerobic L(-)-carnitine production, although titers obtained were lower than under anaerobic conditions (Figure 3).

### Biotransformation with resting cells

**Figure 4:**
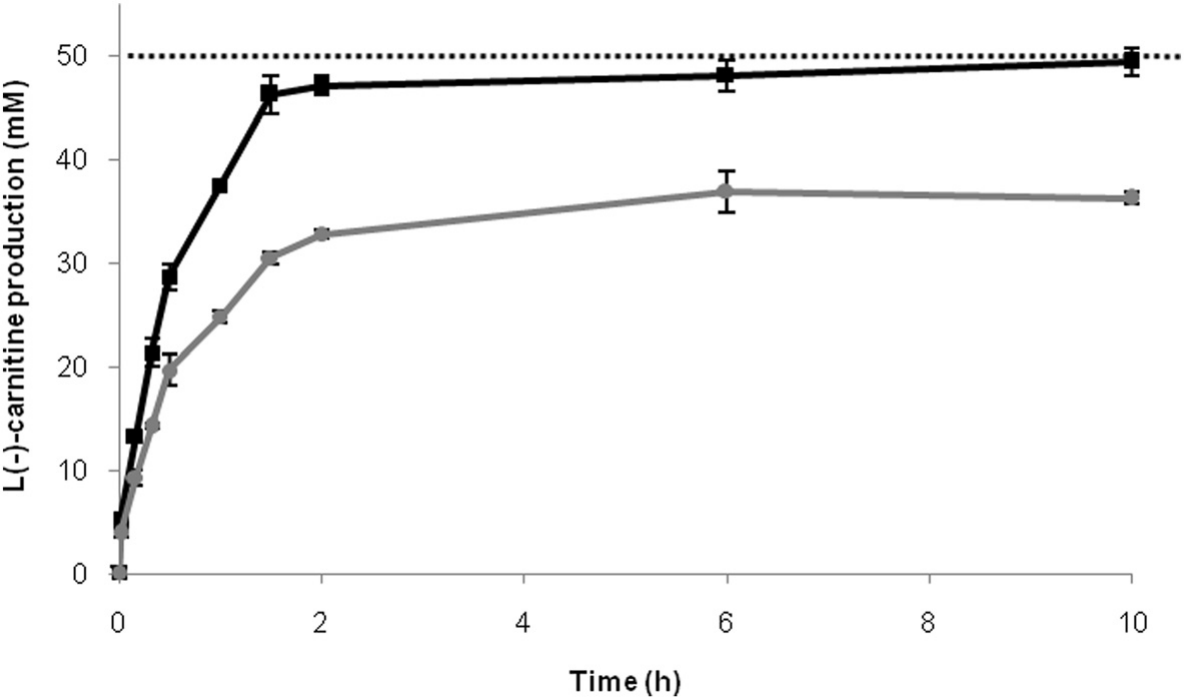
**L(−)-carnitine production by resting cells.** The performance of the BW25113 (wild type) (grey) and BW25113 ∆*aceK* ∆*caiA* p37*cai* (black) strains is compared. Discontinuous line indicates the maximum production (corresponding to 50 mM L(−)-carnitine). Resting cell experiments were performed in phosphate buffered 50 mM crotonobetaine, as explained in the Methods section.

Biotransformation assays were performed with resting cells of the wild type and the BW ∆*aceK* ∆*caiA* p37*cai* strain. Carnitine production was enhanced by resting conditions in both strains. The productivities were 28.5 and 59.6 mmol l^-1^ · h^-1^ for the wild type and the BW ∆*aceK* ∆*caiA* p37*cai* mutant, respectively. The mutant strain reached almost 100% conversion in less than two hours (Figure 4), which is the highest conversion ever reported for L(-)-carnitine producing *E. coli* strains.

### Effect of mutations on control points of the central metabolism

The wild type and the BW ∆*aceK* ∆*caiA* p37*cai* mutant were selected to study the changes imposed by the genetic modifications on central metabolism. To this end, seven metabolites were analyzed: succinate, pyruvate, fumarate, lactate, acetate, ethanol, and formate. Major changes were observed on acetate, which is the main metabolic product and can be considered as readout of the energetic state, and on succinate, which is a product of the mixed acid fermentation pathway and also results from fumarate respiration (Table 2).

In anaerobic cultures supplemented with fumarate, the wild type and the mutant strain exhibited a similar behavior. The maximum concentration of succinate and acetate coincided with the end of the exponential phase, when the culture broth was completely depleted of fumarate (12.2 and 13.9 mM succinate, and 10.2 and 16.4 mM acetate for the wild type and the overproducer strain, respectively). Acetate decreased slightly during the stationary phase. A similar behavior was observed in the assays without fumarate. Formate was only detected in the exponential phase, increasing steeply during the first ten hours of culture, especially in the mutant strain. It was not detected in the stationary phase of cultures, which indicates the activity of formate hydrogen-lyase (Fhl) under these conditions. Changes observed in the fermentation profiles in anaerobic cultures are especially relevant, since they reflect the energy state of cells. Thus, the central metabolism of *E. coli* strains was focused on maintaining suitable levels of ATP and free coenzyme A, which are a limiting factor in the biotransformation process.

The strains grown aerobically showed similar patterns in the metabolite profiles, although differences were found in the levels reached in the stationary phase. At the beginning of the stationary phase, succinate and acetate achieved their maximum concentration (8.3 and 18.1 mM, respectively, for the mutant; 7.4 and 12.5 mM, respectively, for the wild type strain), emphasizing the importance of the acetate metabolism. Moreover, acetate decreased drastically in the later stationary phase, indicating the activity of the acetate scavenging systems [39-41]. Similar acetate profiles were observed in cultures without fumarate. Under aerobic conditions, acetate overflow highlights the need of maintaining the acetyl-CoA/CoASH ratio to meet cellular demands.

## Discussion

This work demonstrates the successful construction of *E. coli* strains engineered for high yielding production of L(-)-carnitine from crotonobetaine. Biotransformation yields ranging from 40 to 95% were obtained in the growing cells system, while conversion was almost complete using resting cells. The strain optimization strategy presented here intended to overcome the major drawbacks previously identified. The modifications introduced had a cumulative effect on strain performance, improving yield and productivity without affecting growth or physiology of the bacteria. In fact, the best results were obtained with the BW ∆*aceK* ∆*caiA* p37*cai* strain which had a growth profile similar to that of the wild type strain.

Previous works demonstrated that the optimization of the biotransformation process depends on primary and secondary metabolisms and its regulation [15,18,34,42,43]. Knockout mutants on the glyoxylate shunt genes *aceK* and *aceA* showed a modest increase in carnitine production [34], underlining the importance of the TCA cycle flux. However, its impact on the productivity was small when compared to the overexpression of enzymes performing the biotransformation such as CaiC and CaiB [23,34,35] or the whole *cai* operon (this work).

The replacement of the promoter of the *caiF* gene with the constitutive p8 promoter enhanced L(-)-carnitine production under aerobic and anaerobic conditions. CaiF is a specific transcriptional activator of carnitine metabolism, binding to two 11-bp inverted repeat half-sites separated by 13 bp in the intergenic region of the two operons *caiTABCDE* and *fixABCX*. The expression of *caiF* is activated by cAMP-CRP and FNR which prevents the expression of the trimethylammonium compounds metabolism in the presence of oxygen and/or glucose [28-30]. Transcriptional repression was overcome after promoter replacement in BW p8*caiF*, although this was not sufficient for optimal performance, especially under aerobic conditions. Various reasons can respond for this observed effect. For instance, although the FNR binding site located at −55.5 bp in the *caiF* promoter was replaced, the presence of further putative half sites for FNR binding is known, which role on *caiF* expression is not known. In addition, the *cai* promoter is also regulated by FNR (two sites at −90.5 and −41.5 bp, respectively) and ArcA (four sites located at −101, -91, +28, and +50 bp) [44], and CaiF overexpression could not be enough for optimal expression under aerobic conditions.

The most remarkable improvement was obtained by tuning the expression of the *cai* operon and deleting *caiA* gene. Replacement of the endogenous promoter by the constitutive promoter p37 enhanced L(-)-carnitine production under anaerobic and aerobic conditions, relieving the *cai* operon from the regulatory effects of oxygen and CaiF. Importantly, the formation of the side-product γ-butyrobetaine was effectively avoided by deleting *caiA*, redirecting crotonobetaine towards L(-)-carnitine production [16,24]. This deletion led to a 25-60% enhancement in L(-)-carnitine production in the BW Δ*caiA* strain and a 30-87% enhancement in the BW Δ*aceK* Δ*caiA* p37*cai* strain. This is the best performing strain obtained, achieving over 95% conversion of the substrate in a growing system, and almost 100% of conversion in a resting cells system.

When the effect of the metabolic engineering strategy on the physiology of the bacteria was assessed, several metabolic changes between the wild type and the BW Δ*aceK* Δ*caiA* p37*cai* strain were observed. Under anaerobic biotransformation conditions, energy producing processes such as fumarate respiration [45,46] and acetate and formate production [40,41,47-50] were enhanced in the mutant. This supports the high dependence of L(-)-carnitine production on the energetic state of cells [15]. Although fumarate was originally used as electron acceptor and inhibitor of the crotonobetainyl-CoA reductase activity (CaiA) [15,24,33], media supplementation with this energetic substrate enhanced growth and L(-)-carnitine production, even in Δ*caiA* strains. Therefore, the reduction of fumarate is a major mechanism of ATP generation in anaerobic conditions [15,42,45], similarly to aerobic oxidative phosphorylation. Increased production of acetate (the end-product of the major energy producing anaerobic pathway of *E. coli*) and formate (the end product of pyruvate-formate lyase) was not observed in aerobic cultures, in which energy is produced by respiration and acetate production occurs as a result of an overflow metabolism [39-41].

It is important to emphasize that the engineered strains created in this work were able to carry out the biotransformation under aerobic conditions, while no L(-)-carnitine was produced by wild type *E. coli*. The best performing strain was BW ∆*aceK* ∆*caiA* p37*cai* with 65% of conversion, a 4-fold higher yield than that reported in previous works under aerobic conditions [33,34]. Fast growth and low biotransformation rate of the BW ∆*aceK* ∆*caiA* p37*cai* strain avoided complete conversion of crotonobetaine. This suggests that other limitations in central metabolism may occur. A plausible explanation for the observed differences between both conditions could be coenzyme A availability, as shown in previous works [15,34,42].

Summarizing, we have successfully engineered *E. coli* for efficient, high-yielding L(-)-carnitine production from an inexpensive substrate (such as crotonobetaine). A stable engineered strain was obtained, which does not depend on expensive inducers (since the p37 and p8 promoters are constitutive) or antibiotics (since all modifications are chromosomal, stable and antibiotic marker-free). In addition, fast transformation was almost complete, therefore with improved downstream processing. Exploitation of this engineered strain in high-density reactors is a feasible and economically viable strategy for the implementation of L(-)-carnitine production processes at the industrial scale.

## Conclusions

L(-)-carnitine production in *E. coli* based growing and resting cells systems has been successfully improved. Multiple stable mutations introduced in a single strain enhanced production without reducing cell viability or affecting specific growth. Furthermore, the biotechnological process was improved and allowed nearly 100% conversion reducing the time of transformation and simplifying downstream processing. Moreover, the main restriction to aerobic expression of the carnitine metabolism was eliminated. This study presents a successful strain improvement strategy by means of gene deletion and promoter replacement and contributes to get further insights into the secondary metabolism of trimethylammonium compounds in *E. coli*.

## Methods

### Strains and plasmids

The wild type strain *E. coli* BW25113 [*lacI*^q^*rrnB*_T14_ ∆*lacZ*_WJ16_*hsdR514* ∆*araBAD*_AH33_ ∆*rhaBAD*_LD78_] was obtained from the Keio collection [51]. The mutant strains constructed in the present study (Table 3) were obtained as described below. The strains were stored in 50% glycerol at −80°C.

**Table 3:** List of bacterial strains used in this work

**Strain**	**References**	**Genotype**	**Short name**
*E. coli* BW25113	Keio collection, Baba et al. [51]	*lacI*^q^*rrnB*_T14_ ∆*lacZ*_WJ16_*hsdR514* ∆*araBAD*_AH33_ ∆*rhaBAD*_LD78_	BW25113
*E. coli* BW25113 ∆*aceK*	This work	[BW25113] ∆*aceK*	BW ∆*aceK*
*E. coli* BW25113 ∆*aceA*	This work	[BW25113] ∆*aceA*	BW ∆*aceA*
*E. coli* BW25113 ∆*aceAK*	This work	[BW25113] ∆*aceAK*	BW ∆*aceAK*
*E. coli* BW25113 ∆*caiA*	This work	[BW25113] ∆*caiA*	BW ∆*caiA*
*E. coli* BW25113 ∆p*caiF caiF*-p8	This work	[BW25113] ∆p*caiF*::*caiF*-p8	BW p8*caiF*
*E. coli* BW25113 ∆p*cai cai*-p37	This work	[BW25113] ∆p*cai*::*cai*-p37	BW p37*cai*
*E. coli* BW25113 ∆*aceK* ∆p*cai cai*-p37	This work	[BW25113] ∆*aceK* ∆p*cai*::*cai*-p37	BW ∆*aceK* p37*cai*
*E. coli* BW25113 ∆*aceK* ∆*caiA* ∆p*cai cai*-p37	This work	[BW25113] ∆*aceK* ∆*caiA* ∆p*cai*::*cai*-p37	BW ∆*aceK* ∆*caiA* p37*cai*

Standard *E. coli* cultures for molecular biology work were performed in Luria-Bertani broth (LB). Antibiotics (ampicillin 100 μg mL^-1^, kanamycin 30 μg mL^-1^, chloramphenicol 30 μg mL^-1^) were added whenever necessary.

The plasmids pKD46 (Red helper plasmid, Ampicillin resistance), pKD3 (containing a FRT-flanked chloramphenicol resistance (*cat*) gene), pKD4 (containing a FRT-flanked kanamycin resistance (*kan*) gene), pCP20 (expressing FLP recombinase activity) [52,53], and pKD-Cre (expressing Cre recombinase activity) were obtained from Prof. Dr. J-P Hernalsteens (Vrije Universiteit Brussels, Belgium). The chloramphenicol resistant (cat) gene flanked by loxP sites and the priming P1 and P2 sites was cloned into pBlueScript using *XbaI* and *BamHI* restrictions sites.

All molecular biology experimentation and strain engineering performed for the completion of this work were approved by the Bioethics Committee of the University of Murcia and complies with all legal requirements.

### Strain engineering: gene knock-out and promoter knock-in strategies

Standard molecular biology protocols were used [54]. Knockout mutants were constructed by successive deletion of specifically targeted genes or regulatory regions using the method of Datsenko and Wanner [53]. Targeted sequences were PCR-amplified using specifically designed primers (see Additional file 1: Table S1) and transformed into pKD46-carrying cells. Mutants were selected for either kanamycin or chloramphenicol resistance. The pCP20-encoding FLP recombinase protein or pKD-Cre-encoding Cre recombinase protein was used to excise the antibiotic-resistance cassette. For the mutation of both *cai/fix* and *caiF* promoters, the promoter knock-in method was used [37]. The specific strategy consisted in the replacement of the respective endogenous promoter sequences by synthetic promoters. Knock-in mutants were constructed from these deletion strains. Two constitutive promoters with different strength were tested for the tuning strategy: p37 (strong) and p8 (weak). These synthetic promoters have been previously described [38] (Figure 2). The mutant strains constructed are listed in Table 3. All constructions were checked by PCR and DNA sequencing.

### Culture conditions

For the biotransformation of L(-)-carnitine from crotonobetaine, a pre-culture was grown at 37°C aerobically using LB medium, pH adjusted to 7.5 with KOH prior to autoclaving (LB). The cultures were inoculated with 3% (v/v) of an overnight grown pre-culture. Cultures were grown under both aerobic and anaerobic conditions at 37°C in LB medium supplemented with 50 mM crotonobetaine as substrate (LB-CB). In some cultures, fumarate 12.5 mM was added acting as electron acceptor and as inhibitor of the reaction catalyzed by the crotonobetainyl-CoA reductase (CaiA). Batch anaerobic assays were performed in 100 mL vessels with 60 mL working volume under nitrogen atmosphere and magnetic stirring. Aerobic assays were performed in 250 mL erlenmeyer flasks with 50 mL working volume in a rotary shaker (150 rpm). The experiments were performed in triplicate.

### Resting cells

For the resting cell assays, anaerobic cultures in LB medium with 5 mM of crotonobetaine, used as inducer of cai operon, were harvested at the end of the exponential growth phase, centrifuged at 16,000xg for 10 min, and washed twice with 67 mM potassium phosphate buffer, pH 7.5. Cells were resuspended in 50 mM potassium phosphate buffer, pH 7.5 with 50 mM crotonobetaine and incubated at 37°C in erlenmeyer flasks in a rotary shaker (150 rpm). All experiments were performed at least in triplicate and under sterile conditions.

### Analytical procedures

Cell growth was followed by optical density (OD) at 600 nm with a spectrophotometer (Novaspec II; Pharmacia-LKB, Sweden) and converted to dry cell weight (DWC). For L(-)-carnitine and extracellular metabolite analysis, cell-free supernatant was obtained by centrifugation at 19,000xg for 10 min. L(-)-carnitine concentration was determined with an enzymatic assay [32].

γ-Butyrobetaine was determined by HPLC [32] with a Spherisorb-NH_2_ column (3 μm, 4.6 × 150 mm) supplied by Waters (Barcelona, Spain). The isocratic mobile phase was acetonitrile/H_3_PO_4_ 0.005 M pH 5.5 (65/35) at a flow rate of 1 mL min^-1^. For the analysis of fermentation products (acetate, ethanol, formate, fumarate, pyruvate, and succinate), a cation exchange Aminex HPX-87H column supplied by BioRad Labs (Hercules, CA) was used. The isocratic mobile phase was 5 mM H_2_SO_4_ at a flow rate of 0.5 mL min^-1^. A HPLC system from Shimadzu (Kyoto, Japan) was used. The effluent was monitored using diode array and refractive index detectors (Shimadzu, Kyoto, Japan).

### RNA isolation and quantitative PCR

RNA was isolated at mid-exponential phase, when L(-)-carnitine production rate was maximum. The cultures were pelleted by centrifugation at 15,000 × *g* at 4°C for 30 s. Total RNA was isolated by Qiagen Rneasy®Mini Kit (QIAGEN Ibérica, Madrid, Spain). Additionally, DNaseI digestion of the isolated RNA was performed using the RNase-free DNase Set (QIAGEN Ibérica, Madrid, Spain) to avoid DNA interferences during PCR steps. RNA quality and quantity were evaluated by microfluidic capillary electrophoresis on an Agilent 2100 Bioanalyzer (Agilent Technologies, PaloAlto, CA) using Agilent RNA 6000 Pico kit. The primers used in this work were designed using the PrimerExpress® Software v3.0 (Applied Biosystems, FosterCity, CA) and ordered from Sigma–Aldrich Co (St. Louis, USA) (see Additional file 1: Table S1). The *dnaA* (encoding the multifunctional initiator of chromosome replication and transcriptional regulator) and *polA* genes (encoding the DNA polymerase I) were used as HKG. Quantitative PCR was performed in a 7300 Real-Time PCR System (Applied Biosystems,Foster City, CA) using PowerSYBR®Green PCR Master Mix (Applied Biosystems, Foster City, CA). Samples were run in triplicate. Raw data were transformed into threshold cycle (Ct) values. Relative gene expression was calculated by the comparative Ct method (∆∆Ct). Experiments were performed in triplicate.

### Statistical analysis of data

The statistical analyses were carried out using R (version 2.15.1). A one-way ANOVA was applied to determine the differences among different conditions and strains. A Tukey test was also carried out to ascertain the significant differences between data pairs. The threshold p-value chosen for statistical significance was p < 0.05.

## Competing interests

The authors have filed a Spanish Patent Application (P201230867), based on part of the results here presented.

## Authors’ contributions

PA carried out the experimental assays and genetic modifications. PA and VB designed the study, analyzed the data and drafted the manuscript. JLI and DC drafted the manuscript. MRFM carried out the design of primers, participated in the genetic modifications and helped to draft the manuscript. MC conceived the study and drafted the manuscript. All authors read and approved the final version of the manuscript.

## Supplementary Material

Additional file 1: Table S1 Primers used for real time PCR. The primers used in this work were designed using the Primer Express® Software v3.0 (Applied Biosystems, Foster City, CA) and ordered from Sigma-Aldrich (Sigma-Aldrich Co., St. Louis, USA). The *dnaA* and *polA* genes (encoding the multifunctional initiator of chromosomal replication and transcriptional regulator and DNA polymerase I, respectively) were used as internal control for relative quantification. Click here for file
