# Supplementary material for: Metabolic engineering for high yielding L(-)-carnitine production in Escherichia coli
Source: Microb Cell Fact. 2013 May 29;12:56. doi: 10.1186/1475-2859-12-56 (PMC3680233; doi:10.1186/1475-2859-12-56)
Supplement: Additional file 1: Table S1 — Primers used for real time PCR. The primers used in this work were designed using the Primer Express® Software v3.0 (Applied Biosystems, Foster City, CA) and ordered from Sigma-Aldrich (Sigma-Aldrich Co., St. Louis, USA). The dnaA and polA genes (encoding the multifunctional initiator of chromosomal replication and transcriptional regulator and DNA polymerase I, respectively) were used as internal control for relative quantification. [file 1475-2859-12-56-S1.doc]

| Gene |  |  |
| --- | --- | --- |
| **Construction of mutant** |  |  |
| Fwd***-****aceAK*-P1 | 5´-CCACATAACTATGGAGCATCTGCACATGAAAACCCGTACACAACGTGTAGGCTGGAGCTGCTTC-3´ | |
| Rev-*aceAK*-P2 | 5´-TTTGCCTGCGCCGATACGCATAAACATCTTCCACATGCCCTTCACCATATGAATATCCTCCTTAG-3´ | |
| Fwd*-caiF*-P1 | 5´-CTGATGAGGACCGTTTTTTTTTGCCCATTAAGTAAATCTTTTGGGGAATCGATATTTGTGTAGGCTGGAGCTGCTTC-3´ | |
| Rev-*caiF*-p8 | 5´-TCTGGCTATGACTCGTTTTGAAGCAATTAAACAAGGCCATATTAAAATTGTGGATTAACCCGCGAATTATATCATATTGG-3´ | |
| Fwd-*caiA-*lox1 | 5´-CTCCCGTAGGCCTGATAAGACGCATCAGCGTCGCATCAGGCAGCGCACGGACATAACTTCGTATAGCATACATTATAC-3´ | |
| Rev-*caiA*-lox2 | 5´-GCGAAACAGAACTGGAAAGATTAATTAACCCCCAAAATATCAAGAGGTTGAAAGATAACTTCGTATAATGTATGCTATACG-3´ | |
| Fwd -*caiTBC*-p37 | 5´-ATTTTTGTTAACATTTAATATAATTATTATTAACCTCGTGGACGCGTTAATGGCAAAAATGACATATACCACATGGA-3´ | |
| Rev-p*caiTBC*-P2 | 5´-ATTTTTTACTCACAACAGAGCATAACAAACTGATTATTAATCAAAATTAACATATGAATATCCTCCTTAG-3´ | |
| **qPCR** | Forward primer | Reverse primer |
| *dnaA* | 5´-TGGCGAAAGAGCTGACTAACC-3´ | 5´-ACGGCAGGCATGAAGCA-3´ |
| *polA* | 5´-GCTGAACGTGCAGCCATTAA-3´ | 5´-CAATCATCGCCCGTTTGATAA-3´ |
| *caiT* | 5´-CACAGTTCACGCACAGTACG-3´ | 5´-GCGCATGTTGTTCTATACCG-3´ |
| *caiB* | 5´-CCGGAAGATTGGTGTACTCC-3´ | 5´-CCGATATCTTCATCGAAGCC-3´ |
| *caiC* | 5´-GGCGACAAGGTTGCACTACA-3´ | 5´-AGCCCGAACCAGCAAAAGA-3´ |
| *fixA* | 5´-GCAGGCTTTGATCTGATCCT-3´ | 5´-CTCAACGGTGAGGGTATCTG-3´ |

**Additional file 1: Table S1. Primers used for real time PCR**. The primers used in this work were designed using the Primer Express® Software v3.0 (Applied Biosystems, Foster City, CA) and ordered from Sigma-Aldrich (Sigma-Aldrich Co., St. Louis, USA). The dnaA and polA genes (encoding the multifunctional initiator of chromosomal replication and transcriptional regulator and DNA polymerase I, respectively) were used as internal control for relative quantification.
